# Poricoic Acid A from *Poria cocos* Attenuates AGE-Induced Podocyte Injury: Network Pharmacology Analysis and In Vitro Validation

**DOI:** 10.3390/nu18203432

**Published:** 2026-10-19

**Authors:** Xia-Qing Wu, Ling-Ling Li, Hui Wang, Huan-Le Fang, Fang-Jian Ning

**Affiliations:** 1 School of Medical, Xi’an Peihua University, No. 888 Changning Road, Xi’an 710125, China; wxq-607697@163.com (X.-Q.W.);; 2 Key Laboratory of Geriatric Nutrition and Health, School of Food and Health, Beijing Technology and Business University, Ministry of Education, No. 33 Fucheng Road, Beijing 100048, China

**Keywords:** medicine–food homology, *Poria cocos*, poricoic acid A, advanced glycation end product, podocyte injury

## Abstract

Background/Objectives: *Poria cocos* (PC), a well-known medicine–food homologous fungus, has promising reno-protective potential against diabetic kidney disease (DKD); however, its bioactive constituents and underlying molecular mechanisms remain poorly elucidated. This study aimed to decipher the reno-protective potential of PC via network pharmacology, and to subsequently validate the cyto-protective effect of poricoic acid A (PAA), a major active component of PC, against advanced glycation end product (AGE)-induced podocyte injury. Methods: Network pharmacology was adopted to identify active ingredients, core targets and pivotal pathway of PC. Molecular docking was performed to analyze binding interactions between the identified active ingredients and core targets. The AGE-induced podocyte model was used to detect cell viability, mRNA and protein expression. Results: Network analysis identified that nine active ingredients, five core targets (*PIK3CA*, *AKT1*, *TNF*, *MAPK3*, and *MAPK8*), and pivotal signaling pathways, including the AGE/RAGE signaling pathway, proteoglycans in cancer, etc., constitute key pharmacological nodes of PC’s bioactivity. Molecular docking suggested that PAA exhibits favorable binding affinity toward these five key targets. In cellular assays, 100 μg/mL PAA improved cell viability in AGE-induced podocytes. Mechanistically, PAA down-regulated *RAGE* mRNA expression; suppressed the phosphorylation of PI3K, AKT, and NF-κB; and decreased the levels of NLRP3 inflammasome-related proteins. Conclusions: Collectively, this study first reveals the reno-protective potential of the PC-derived bioactive component PAA in mitigating AGE-induced podocyte injury, which may be associated with the regulation of RAGE-PI3K/AKT and RAGE/NLRP3 signaling axes. These findings provide theoretical evidence at the cellular level for further exploring PC-based functional foods against AGE-induced podocyte injury in DKD.

## 1. Introduction

Diabetic kidney disease (DKD) is the most common complication of diabetes, affecting approximately 20% to 40% of diabetic patients worldwide [1]. The International Diabetes Federation Diabetes Atlas (10th edition) reported that about 537 million adults aged 20–79 years in the world are diagnosed with diabetes, and this number is projected to reach 643 million by 2030 and 783 million by 2045, respectively [2]. Currently, the clinical management of DKD primarily focuses on metabolic control, using medications such as metformin and angiotensin receptor blockers to ameliorate hyperglycemia, hypertension, and hyperlipidemia. However, significant side effects, including gastrointestinal intolerance associated with metformin and recurrent hypoglycemia induced by intensive glucose-lowering therapy, compromise the clinical outcomes of DKD treatment [3].

Accumulating evidence has demonstrated that the excessive generation and accumulation of advanced glycation end products (AGEs) in the kidney is closely related to DKD pathogenesis [4]. AGEs are highly active, irreversible products formed by non-enzymatic glycation reactions between reducing sugars (like glucose) and biomacromolecules (including proteins, lipids, and nucleic acids) [5]. Chronic hyperglycemia under diabetic conditions accelerates glycation processes, promoting the overproduction and deposition of AGEs, subsequently activating the receptor for AGE (RAGE). AGE-RAGE interaction further triggers multiple intracellular signaling pathways, such as PI3K/AKT, MAPK/ERK, and NF-κB, by activating key downstream mediators including NF-κB, PIK3CA, and MAPK [6,7]. Consequently, targeting the AGE/RAGE axis seems to be a promising therapeutic strategy for intervening in DKD.

Growing attention has been paid to natural food–medicine homologous ingredients for preventing metabolic diseases due to their high safety and multi-target regulatory properties [8,9]. *Poria cocos* (PC) is a typical food–medicine homologous herb possessing diuretic and reno-protective properties. It is widely applied in diverse herbal formulas, such as Chinese herbal prescriptions, traditional formulas, and chemically modified preparations, for improving the management of kidney diseases [10,11,12]. Recent studies have confirmed that PC exerts beneficial effects in diabetes and nephrotic syndrome [13]. High-dose PC as well as PC-containing compound formulas were confirmed to effectively relieve DKD [14]. In terms of bioactive constituents, trametenolic acid was reported to ameliorate DKD progression in db/db mice by regulating Nrf2/HO-1 and NF-κB signaling cascades [15]. Furthermore, poricoic acid A (PAA) has been shown to alleviate high glucose–induced podocyte injury and inhibit renal fibrosis in DKD mice by regulating the FUNDC1-related pathway [16,17]. Despite these findings, the core active components, pivotal targets, and precise molecular mechanisms underlying the reno-protective effect of PC against DKD remain unclear. Network pharmacology offers a systematic approach to investigate these challenges by extracting target genes associated with herbal active components and disease phenotypes, followed by network topology analysis [18]. This strategy is well-suited to dissect the material basis and molecular mechanisms underlying the protective effects of PC in DKD.

This study aims to identify the bioactive components, therapeutic targets, and molecular mechanisms of PC against DKD, with a focus on evaluating the pharmacological efficacy of its primary active constituent PAA. First, network pharmacology was utilized to screen the active compounds of PC, the core therapeutic targets, and key signaling pathways involved in DKD treatment. Subsequently, molecular docking analysis was performed to assess the interaction modes and binding affinity between the top five core targets and potential active compounds of PC. Finally, an AGE-induced MPC5 cell injury model was adopted to simulate the pathological state of DKD in vitro and verify the protective effects of PAA. This study aims to provide novel mechanistic insights and experimental evidence for PC-derived PAA by exploring its potential protective effects against podocyte injury in the setting of DKD.

## 2. Materials and Methods

### 2.1. Chemicals and Reagents

Mouse podocyte clone 5 (MPC5) cells were purchased from the China Center for Type Culture Collection. AGE (A8301) was procured from Sigma-Aldrich (St. Louis, MO, USA), its preparation and characterization were performed by the manufacturer. PAA (B25591) and RIPA lysis buffer (R21237) were supplied by Yuanye Biotechnology Co., Ltd. (Shanghai, China). Cell Counting Kit-8 (CCK-8, KGA317) was obtained from KeyGen Biotech (Nanjing, China). Pierce™ BCA Protein Assay Kit (23227) and ECL reagent (34577) were provided by Thermo Fisher Scientific Inc. (Waltham, MA, USA). 30% Acr-Bis (A1026), SDS (S8010), pH 6.8 Tris-HCl buffer (1 mol L^−1^, T1020) and pH 8.8 Tris-HCl buffer (1.5 mol L^−1^, T1010), and PMSF (P0100) were obtained from Solarbio Science and Technology Co., Ltd. (Beijing, China). TEMED(T105496) and APS (A112450) were supplied by Aladdin Scientific (Shanghai, China).

### 2.2. Identification of Potential Therapeutic Targets of PC Against DKD

The active ingredients of PC were retrieved from the Traditional Chinese Medicine Systems Pharmacology (TCMSP) platform. Compounds satisfying oral bioavailability (OB) ≥ 30% and drug-likeness (DL) ≥ 0.18 were selected for preliminary screening. These cut-off values are default parameters derived from the classic TCMSP database and are widely adopted in recent studies [19,20]. Subsequently, the screened compounds were imported into PubChem to retrieve their canonical SMILES strings and PubChem compound identifiers (CIDs), and the CIDs served as unique compound identifiers. The obtained SMILES were then submitted to the SwissTargetPrediction database to predict the potential targets of these compounds, with the species set to *Homo sapiens*. Targets with a credibility value of 0 were excluded to ensure prediction reliability.

All database retrievals in this study were completed on 20 September 2025. DKD-related targets were collected from GeneCards and DisGeNET, with the search terms “diabetic kidney” and “diabetic nephropathy”. For GeneCards, 977 genes with a relevance score above the median value were retained from 18820 raw entries. For DisGeNET, 594 DKD-associated genes with GDA-score above the median value were kept from 1189 raw entries. The overlapping targets between active ingredients in PC and DKD were identified as the potential therapeutic targets of PC against DKD.

### 2.3. Construction of Protein–Protein Interaction (PPI) Network and Screening of Core Targets

The overlapping targets were obtained by Venny software and were further imported into the STRING database to construct a PPI network. The protein type was set to *Homo sapiens*, and the minimum interaction confidence score was defined as 0.4. The raw data of protein interactions were loaded into Cytoscape (v3.8.2) to calculate network topological parameters. A refined PPI map was reconstructed based on betweenness centrality, closeness centrality, and degree value. The hub targets were finally screened using the median degree value as the filtering threshold.

### 2.4. GO Function and KEGG Pathway Enrichment Analysis

The hub targets were subjected to GO and KEGG enrichment analyses using the DAVID database. The gene identifier restriction was “Official_Gene_Symbol”, and the species was limited to “*Homo sapiens*”. The enrichment data covering GO functional terms and KEGG pathways were acquired. For GO enrichment, the top biological terms with significant *p*-values in biological processes (BPs), cellular components (CCs), and molecular functions (MFs) were selected and visualized in bar charts. In the KEGG enrichment analysis, the top 20 pathways meeting the significance criterion were screened and then presented as bubble plots.

### 2.5. Construction and Analysis of Component–Target–Pathway Network

The topological network of component–target–pathway interactions was constructed using Cytoscape. Three topological network parameters, including degree, betweenness centrality, and closeness centrality, were calculated for each node. Nodes with at least two parameter values greater than or equal to the respective median were identified as critical nodes, which represent key compounds, core targets, or pathways.

### 2.6. Molecular Docking Simulation

Molecular docking between the active ingredients and core targets was conducted using AutoDock Vina 1.1. The screened active ingredients comprised FL1, FL2, FL4, FL8, FL9, FL10, FL11, FL13, and FL15. Full information on the 15 candidate compounds (including FL label, Mol ID, molecule name, PubChem CIDs, etc.) is listed in Supplementary Table S1. (1): (2R)-2-[(3S,5R,10S,13R,14R,16R,17R)-3,16-dihydroxy-4,4,10,13,14-pentamethyl-2,3,5,6,12,15,16,17-octahydro-1H-cyclopenta[a]phenanthren-17-yl]-6-methylhept-5-enoic acid; (2): trametenolic acid; (3): cerevisterol; (4):(2R)-2-[(5R,10S,13R,14R,16R,17R)-16-hydroxy-3-keto-4,4,10,13,14-pentamethyl-1,2,5,6,12,15,16,17-octahydrocyclopenta[a]phenanthren-17-yl]-5-isopropyl-hex-5-enoic acid; (5): 3beta-Hydroxy-24-methylene-8-lanostene-21-oic acid; (6): pachymic acid; (7): poricoic acid A; (8): poricoic acid C; (9): dehydroeburicoic acid. Based on binding energy values, the top five core targets were identified as *PIK3CA*, *AKT1*, *TNF*, *MAPK3*, and *MAPK8*.

The chemical structures of active ingredients were retrieved from the PubChem database, followed by force-field optimization and hydrogen atom addition. The crystal structures of the target proteins were obtained from the Protein Data Bank, and the PDB IDs used were 6GVF, 4EKL, 1A8M, 6GES, and 3PZE, respectively. All target proteins were prepared by removing water molecules, adding polar hydrogens, and repairing missing residues. For docking-box definitions, the box dimension was fixed at 22.5 × 22.5 × 22.5 Å for all targets. The center coordinates for each individual protein were as follows: 6GVF (*x* = −16.6, *y* = 147.0, *z* = 28.4); 4EKL (*x* = 28.0, *y* = 5.2, *z* = 10.9); 1A8M (*x* = 9.0, *y* = 62.6, *z* = 30.4); 6GES (*x* = 61.3, *y* = 21.1, *z* = 18.5); and 3PZE (*x* = 21.6, *y* = 9.6, *z* = 31.8). Protein–ligand systems were prepared under the physiological condition of pH 7.4. The num_modes parameter was set to 20, which controls the maximum number of candidate binding poses per docking run. The co-crystallized ligand-receptor complex (PDB ID: 4EKL) was selected as a representative system for redocking validation. The validation was performed by using the above-mentioned docking parameters together with the center coordinates of 4EKL to confirm the applicability of this docking protocol for this study. The RMSD between the top-ranked redocked pose and the native co-crystallized ligand was 1.8 Å. The protein–ligand interactions were analyzed using the Protein–Ligand Interaction Profiler. Docking poses with more negative binding affinities were considered the most stable molecular conformations and were visualized by PyMOL(v2.2.2).

### 2.7. In Vitro Experiments Using MPC5 Cells

#### 2.7.1. Cell Viability Assay

MPC5 cells were cultured using the method described by Li et al. [21]. In brief, cells were seeded into 96-well plates at a density of 5 × 10^3^ cells/well and were cultured in an incubator at 37 °C with 5% CO_2_. When the density of the cells reached approximately 60% confluence, PAA solution at varying concentrations (25, 50, 100, 150, and 200 μg/mL) or AGE solution at different concentrations (100, 150, 200, 250, and 300 μg/mL) was added to replace the original medium. After an additional 24 h of culture, cell viability was assessed using the CCK-8 assay. Specifically, CCK-8 reagent was added to each well and incubated for 2 h. The absorbance at 450 nm was detected by a microplate reader (SuPerMax 3100, Shanghai Shanpu, China). Cell viability was calculated from the absorbance, and the formula is as follows: Cell viability (%) = (*A*_s_ − *A*_b_)/(*A*_c_ − *A*_b_) × 100%, where *A*_s_, *A*_c_, and *A*_b_ represent the absorbance of the experimental wells, control wells, and blank wells, respectively.

#### 2.7.2. AGE-Induced MPC5 Cells Treated with PAA

MPC5 cells were seeded and cultured as described above until 60% confluence, and then cells were treated with 200 μg/mL of AGE for 24 h, in the presence or absence of 100 μg/mL PAA. Cell viability was calculated as described in Section 2.7.1.

#### 2.7.3. Quantitative Real-Time PCR (qRT-PCR) Analysis

The mRNA expression of *RAGE* was determined using qRT-PCR, as previously described by Zhang et al. [22]. In brief, total RNA in MPC5 cells was extracted using the TransZol Up Plus RNA Kit according to the manufacturer’s instructions (ER501-01, TransGen Biotech, Beijing, China). The reverse transcription procedure was performed according to the *TransScript*^®^ Uni All-in-One First-Strand cDNA Synthesis SuperMix for qPCR (AU341, TransGen Biotech, Beijing, China). A pre-mixed system of MagicSYBR Mixture (CW3008, Cwbiotech, Beijing, China) was used for qRT-PCR. The data were normalized to the housekeeping gene *β*-actin, and the fold change in gene expression was analyzed using the 2^−ΔΔ*Ct*^ relative quantification method. The primers were synthesized by Shanghai Sangon Biotech Co., Ltd (Shanghai, China). The sequences were as follows: RAGE forward 5′-GCCACTGGAATTGTCGATGAGG-3′ and reverse 5′-GCTGTGAGTTCAGAGGCAGGAT-3′; GAPDH forward 5′-CATCACTGCCACCCAGAAGACTG-3′ and reverse 5′-ATGCCAGTGAGCTTCCCGTTCAG-3′. The PCR procedure was conducted under the following conditions: preliminary denaturation at 95 °C for 10 min, followed by 95 °C for 10 s, 60 °C for 30 s, and 72 °C for 30 s.

#### 2.7.4. Western Blot

MPC5 cells, including controls, AGE-induced cells, and AGE-induced MPC5 cells treated with PAA, were lysed using RIPA lysis buffer supplemented with PMSF. The lysates were placed on ice for 15 min and subsequently centrifuged at 12,000× *g* for 15 min at 4 °C. Protein concentrations in the supernatants were determined using the BCA method.

Equal amounts of protein were separated by 10% SDS-PAGE and then transferred onto PVDF membranes. The membranes were blocked with blocking solution (P0252, Beyotime, Shanghai, China) for 1 h, followed by washing with PBS. Immunoblotting was performed using primary antibodies against p-PI3K (1:1000, ab182651, Abcam, Cambridge, MA, USA), PI3K (1:5000, ab40776, Abcam, Cambridge, MA, USA), p-NF-κB (1:2000, ab16502, Abcam, Cambridge, MA, USA), NF-κB (1:3000, 10745-1-AP, Proteintech, Wuhan, China), p-AKT (1:800, AF0016, Cohesion, Suzhou, China), AKT (1:800, CQA8001, Cohesion, Suzhou, China), NLRP3 (1:1000, 15101, CST, Danvers, MA, USA), caspase-1 (1:800, 22915-1-AP, Proteintech, Wuhan, China), GSDMD (1:1000, 39754, CST, Danvers, MA, USA), ASC (1:1000, 67824, CST, Danvers, MA, USA), and *β*-actin (1:2000, 20536-1-AP, Proteintech, Wuhan, China). The membranes were incubated with the primary antibodies overnight at 4 °C, washed with PBS, and then incubated with HRP-labeled goat anti-rabbit secondary antibody (1:10,000, SA00002-1, Proteintech, Wuhan, China) or HRP-labeled goat anti-mouse secondary antibody (1:10,000, SA00002-2, Proteintech, Wuhan, China) for 2 h at room temperature. Protein bands were visualized using ECL reagent. Band intensities of target proteins were quantified by ImageJ (v1.54), with the operator blinded to experimental information. Phosphoprotein signal intensities were normalized to their respective total protein signals, where total protein signals were pre-normalized to the internal reference *β*-actin. Pathway activation was evaluated using the phosphoprotein/total protein signal ratio.

### 2.8. Statistical Analysis

Experimental data were presented as the mean ± standard error of the mean (SEM). Three independent biological replicates were performed for all experiments. For each biological replicate, technical replicate values were averaged before the statistical analysis. Statistical analyses were performed using GraphPad Prism software (v8.2.1). One-way analysis of variance (ANOVA) was applied to assess the overall differences among groups, followed by Tukey’s post-hoc multiple comparisons test. All exact adjusted *p*-values were provided in Supplementary Table S2. A *p*-value of less than 0.05 was considered statistically significant, while *p*-values of less than 0.01 and 0.001 were highly significant. The corresponding significance symbols were indicated in the Figures.

## 3. Results

### 3.1. Target Prediction and Identification of PC Against DKD

Network pharmacology analysis was employed to investigate the potential targets of PC against DKD. A total of 15 active ingredients (designated F1-F15) of PC were retrieved from the TCMSP database, corresponding to 872 putative targets (Supplementary Table S3). Notably, certain targets were shared among multiple ingredients, suggesting that these compounds may exert synergistic biological effects in vivo. After removing duplicates, a total of 249 predicted targets of PC were identified.

**Figure 1:**
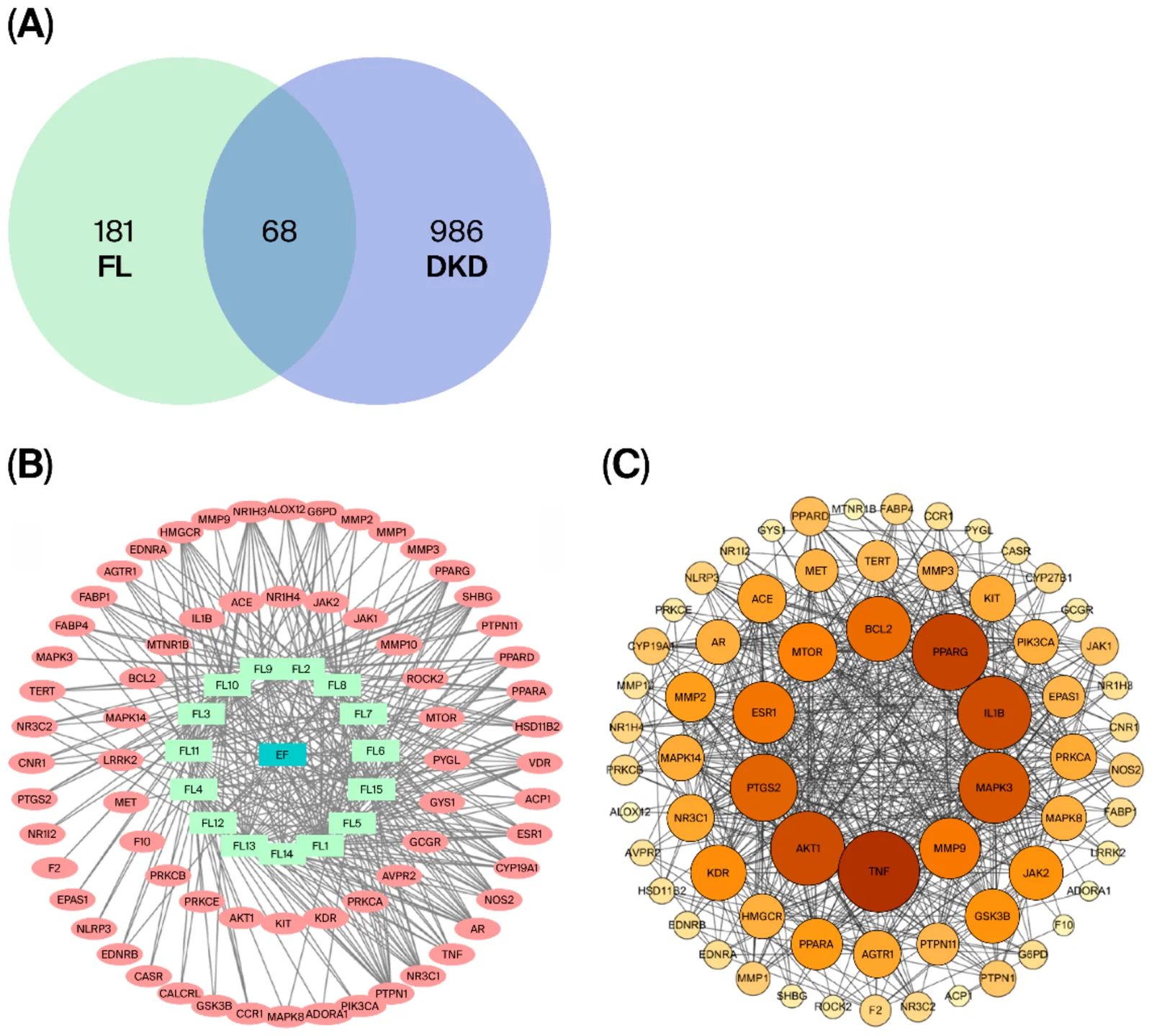
Target analysis of *Poria cocos* (PC) treating diabetic kidney disease (DKD). (**A**) Venn diagram illustrating the overlapping targets between PC and DKD. (**B**) Compound–target network diagram composed of the effective ingredients in PC and the overlapping targets. Red ellipses and green rectangles denote the predicted targets and bioactive compounds in PC, respectively. The lines indicate interactions between compounds and targets. (**C**) Protein–protein interaction (PPI) network of core hub targets. Node size and color gradient are proportional to degree values: larger and darker brown nodes indicate higher degrees, while smaller light-yellow nodes indicate lower degrees.

A total of 1571 DKD-related targets were obtained from GeneCards and DisGeNET; after eliminating 517 redundant entries, the remaining targets were considered for further analysis (Supplementary Table S3). The intersection of PC and DKD targets was visualized using a Venn diagram (Figure 1A), revealing 68 common targets (Supplementary Table S3). The “Effective drug–active ingredient–potential target” network, comprising 1 EF(PC), 15 compounds, and 68 targets, is shown in Figure 1B.

### 3.2. Construction of PPI Network and Identification of Core Targets

To explore the potential mechanism of PC against DKD, a PPI topological network was constructed based on the 68 common targets. As shown in Figure 1C, the network consisted of 68 nodes and 2250 edges, with node sizes proportional to the degree value of each target. The targets with a degree value greater than or equal to 15 were designated as hub targets of PC treating DKD. In total, 34 hub targets were screened from the PPI network.

### 3.3. GO and KEGG Pathway Analysis

**Figure 2:**
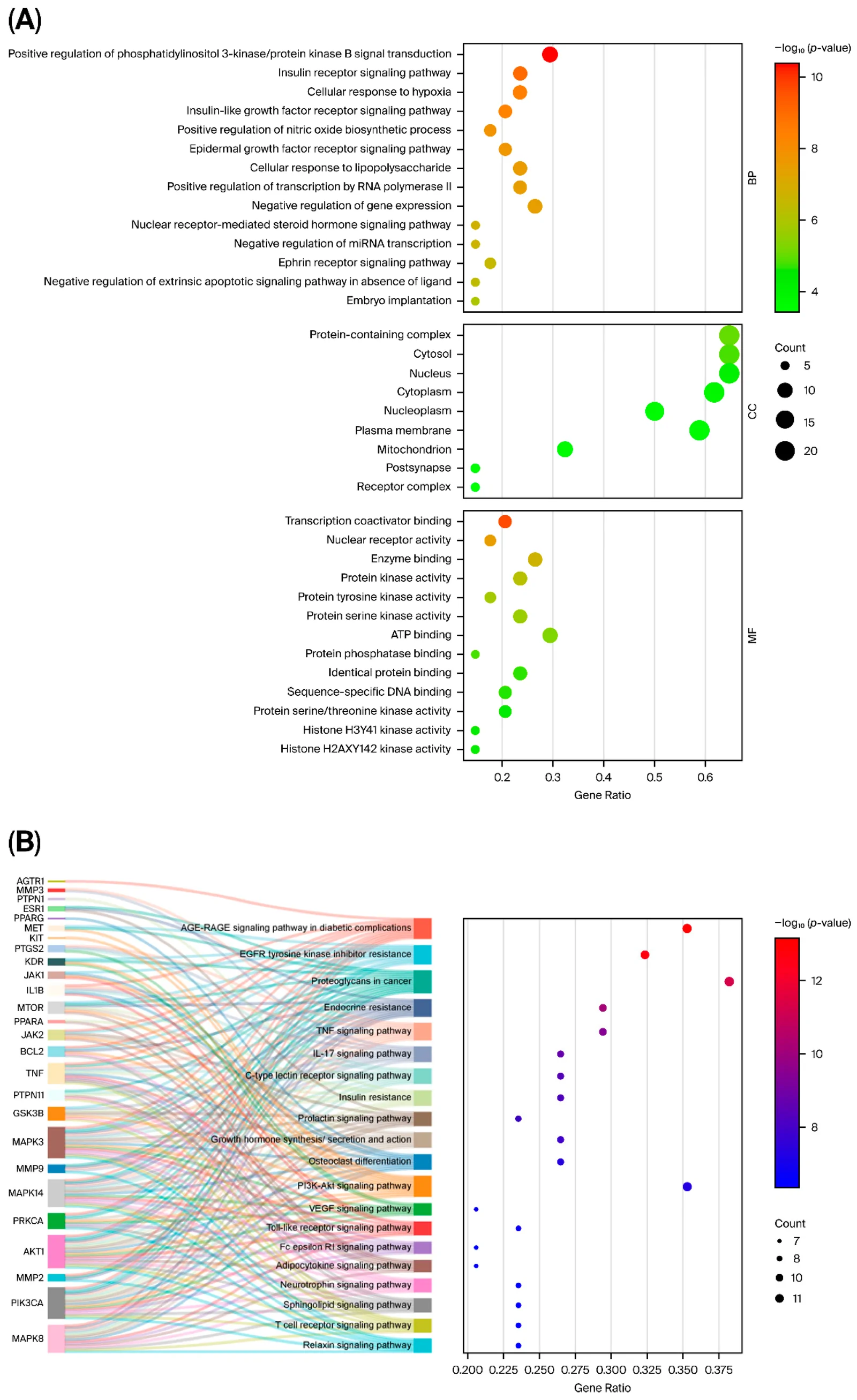
GO and KEGG functional enrichment analyses of core targets. (**A**) GO enrichment analysis. The *x*-axis represents the gene ratio, and the *y*-axis lists enriched terms categorized into biological processes (BPs), cellular components (CCs), and molecular functions (MFs). (**B**) Combined Sankey-dot plot of KEGG pathway enrichment. In the Sankey diagram (**left**), the left-side rectangles represent targets, the right-side rectangles represent pathways, and the connecting lines indicate the associations between each target and its corresponding pathways. The dot plot (**right**) shows the enrichment significance [−log_10_(*p*-value)] and gene ratio of each pathway.

To systematically elucidate the potential mechanism of PC treating DKD, GO enrichment analysis based on the 34 hub targets was performed. The results indicated that these targets were enriched in 55 BPs, 28 CCs, and 101 MFs. The top 14 BPs, 9 CCs, and 13 MF terms, ranked by −log_10_(*p*-values), are shown in Figure 2A. According to the highest −log_10_(*p*-value) rankings, the enriched BP terms were primarily involved in the positive regulation of phosphatidylinositol 3-kinase/protein kinase B signal transduction and the insulin receptor signaling pathway. The major CC terms included protein-containing complex and cytosol, while the predominant MF terms comprised transcription coactivator binding, nuclear receptor activity, and enzyme binding.

KEGG enrichment analysis was conducted to predict the signaling pathways associated with the potential targets of PC treating DKD. A total of 138 signaling pathways were identified, with the top 20 enriched pathways ranked by −log_10_(*p*-values) (*p* < 0.01) demonstrated in Figure 2B. Key pathways included the AGE-RAGE signaling pathway, EGFR tyrosine kinase inhibitor resistance, proteoglycans in cancer, etc.

### 3.4. Regulatory Construction of Compound–Target–Pathway Network

**Figure 3:**
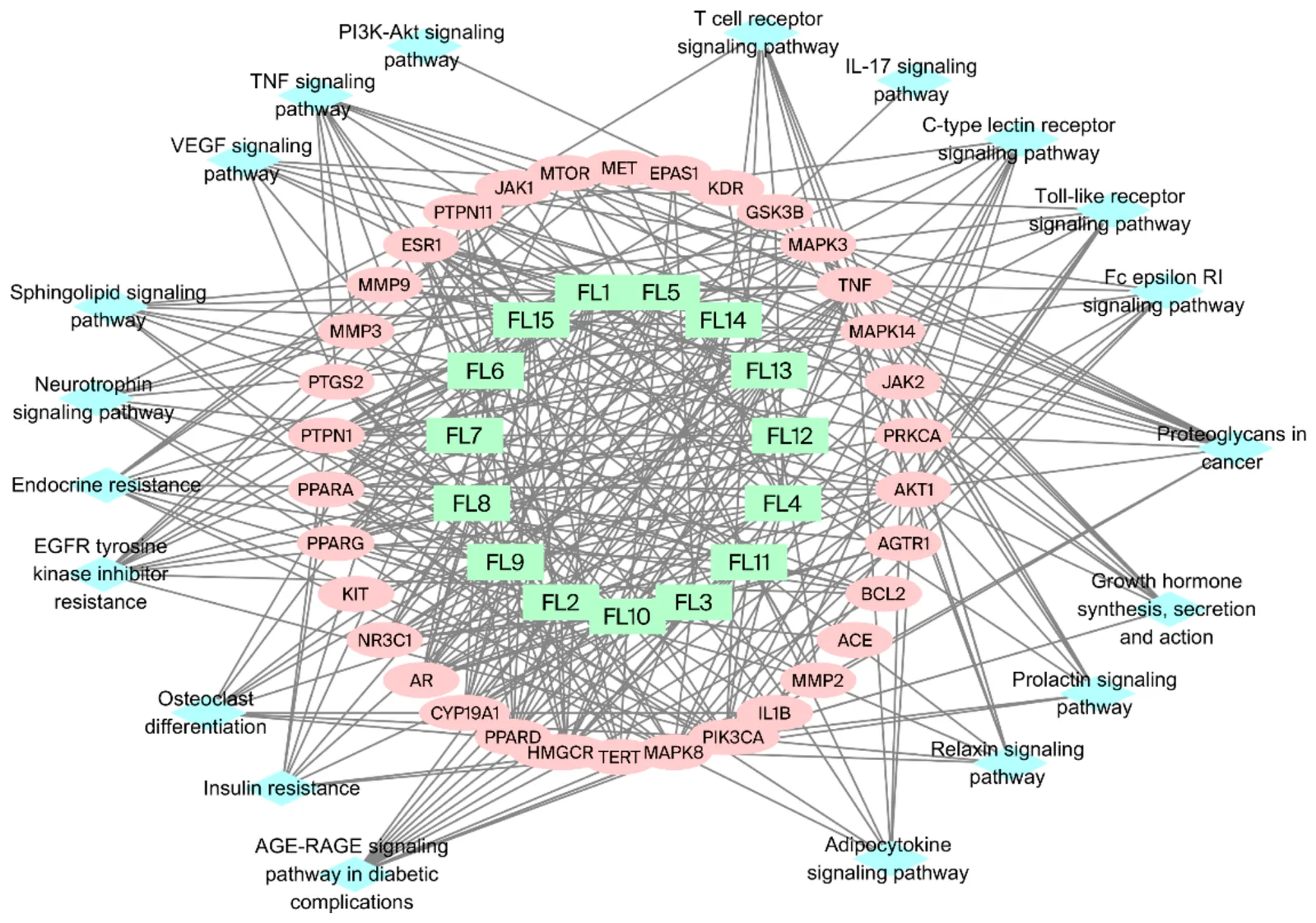
Compound–target–pathway network illustrating relationships among bioactive compounds of PC, targets, and enriched KEGG pathways. Cyan parallelograms denote signaling pathways, red ellipses represent potential targets, and green rectangles indicate bioactive compounds.

The compound–target–pathway network was constructed to identify the core targets, potential compounds, and key pathways. As shown in Figure 3, the network comprises 68 nodes and 2400 edges. FL1, FL2, FL4, FL8, FL9, FL10, FL11, FL13, and FL15 were found to be active compounds derived from PC, and the number of targets is 17, 13, 16, 10, 13, 13, 13, 14, and 13, respectively. The top five targets, *PIK3CA*, *AKT1*, *TNF*, *MAPK3*, and *MAPK8,* were associated with 19, 19, 19, 18, and 17 pathways, respectively (Table 1). The potential binding modes and binding affinities between these core targets and active compounds were further evaluated.

### 3.5. Molecular Docking Analysis

**Figure 4:**
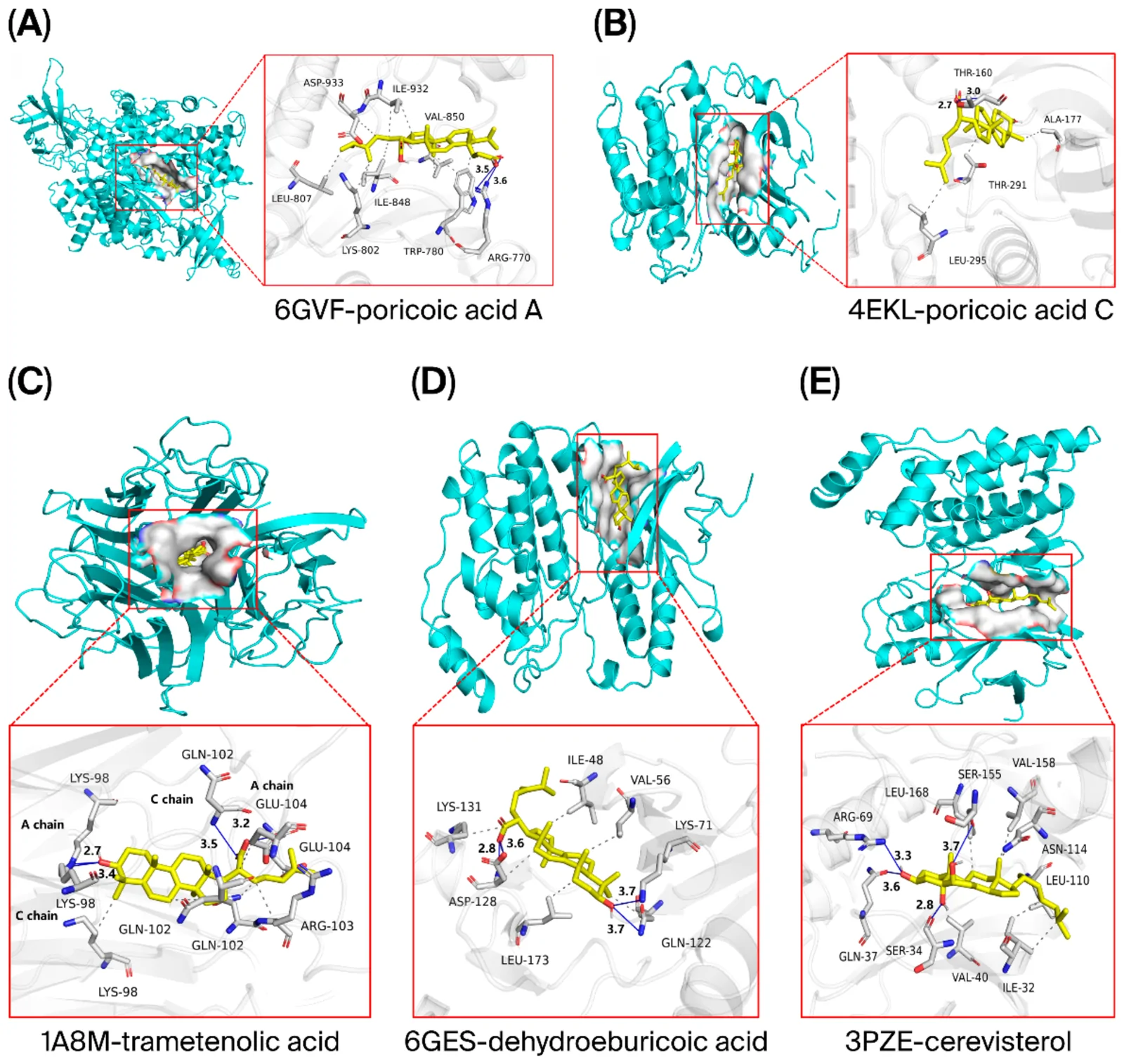
3D and 2D interaction diagrams of representative docking complexes. Each panel (**A**–**E**) shows one compound–protein complex with its 3D binding conformation and 2D intermolecular interactions. 6GVF(PIK3CA)-poricoic acid A (**A**); 4EKL(AKT1)-poricoic acid C (**B**); 1A8M(TNF)-trametenolic acid (**C**); 6GES(MAPK3)-dehydroeburicoic acid (**D**); 3PZE(MAPK8)-cerevisterol (**E**).

AutoDock Vina, a widely used docking program, was employed to estimate binding affinities [23]. A more negative binding energy indicates a potential binding tendency between the active ingredient and its target, but this does not represent actual intermolecular interactions in a biological system. As shown in Table 2, the active compounds (PAA, poricoic acid C, trametenolic acid, dehydroeburicoic acid, and cerevisterol) exhibited favorable binding to their respective optimal target proteins (6GVF, 4EKL, 1A8M, 6GES, 3PZE), with binding energies of −7.5, −9.0, −5.1, −8.0, and −8.0 kcal/mol, respectively. The 3D docking models (Figure 4) clearly demonstrated that the active compounds were embedded within the binding cavities of their target proteins and established interactions with some specific amino acid residues. The 2D interaction diagrams further illustrated these binding details. Specifically, PAA interacted with key residues within the binding pocket of 6GVF(PIK3CA), including ARG770, TRP780, LYS802, LEU807, ILE848, VAL850, ILE932, and ASP933. Among these, two conventional hydrogen bonds were formed between the O2 and O3 atoms of PAA and the guanidinium group of ARG770, with bond distances of 3.5 and 3.6 Å, respectively (Figure 4A). Additionally, hydrophobic interactions involving TRP780, LYS802, LEU807, ILE848, VAL850, ILE932, and ASP933 were observed, further stabilizing the PAA-target complex.

Poricoic acid C exhibited potential interactions with amino acid residues within the binding pocket of 4EKL(AKT1), including THR160, ALA177, THR291, and LEU295. Two conventional hydrogen bonds were formed between the O3 atom of poricoic acid C and THR160, involving the main-chain amide nitrogen and the side-chain hydroxyl oxygen, with bond distances of 3.0 and 2.7 Å, respectively (Figure 4B).

Trametenolic acid exhibited binding interactions with amino acid residues within the binding pocket of 1A8M (TNF), involving LYS98 from chains A and C, GLN102 from chain C, and GLU104 from chain A. Four hydrogen bonds were identified (Figure 4C). Specifically, two hydrogen bonds were formed between the side-chain ammonium groups of LYS98 from chains A and C and the same O3 atom of trametenolic acid, with bond distances of 2.7 and 3.4 Å, respectively. An additional hydrogen bond was established between the main-chain amide nitrogen of GLN102 (chain C) and a distinct O3 atom, with a bond distance of 3.5 Å. A fourth hydrogen bond was observed between the side-chain carboxylate oxygen of GLU104 (chain A) and another O3 atom, at a bond distance of 3.2 Å.

Dehydroeburicoic acid has interactions with amino acid residues within the binding pocket of 6GES (MAPK3), including ILE48, VAL56, LYS71, GLN122, ASP128, LYS131, and LEU173. A total of four hydrogen bonds were identified (Figure 4D). Two hydrogen bonds were formed with the same O3 atom of dehydroeburicoic acid: one involving the side-chain ammonium group of LYS71 and the other involving the side-chain amide nitrogen of GLN122, with both bond distances of 3.7 Å. A third hydrogen bond was established between the main-chain amide nitrogen of ASP128 and a separate O3 atom, at a bond distance of 2.8 Å. A fourth hydrogen bond was observed between the side-chain carboxylate oxygen of ASP128 and the O^-^ atom of the compound, with a bond distance of 3.6 Å.

Cerevisterol had potential interactions with amino acid residues within the binding pocket of 3PZE (MAPK8), including ILE32, SER34, GLN37, VAL40, ARG69, LEU110, ASN114, SER155, VAL158, and LEU168. A total of four hydrogen bonds were observed (Figure 4E). Specifically, one hydrogen bond was formed between the side-chain hydroxyl oxygen of SER34 and an O2 atom of cerevisterol, with a bond distance of 2.8 Å. Another hydrogen bond was established between the side-chain amide nitrogen of GLN37 and a distinct O2 atom, at a bond distance of 3.6 Å. A third hydrogen bond was observed between the guanidinium group of ARG69 and the O3 atom, with a bond distance of 3.3 Å, and a fourth hydrogen bond was formed between the side-chain hydroxyl oxygen of SER155 and another O2 atom, at a bond distance of 3.7 Å.

Collectively, hydrogen bonds were identified as the predominant non-covalent interaction force stabilizing these complexes. PAA, the primary compound in PC, was selected to explore its potential modulatory effect on the AGE-RAGE axis in the injured MPC5 cells.

### 3.6. Cell Experiments

#### 3.6.1. Optimal PAA Concentration

**Figure 5:**
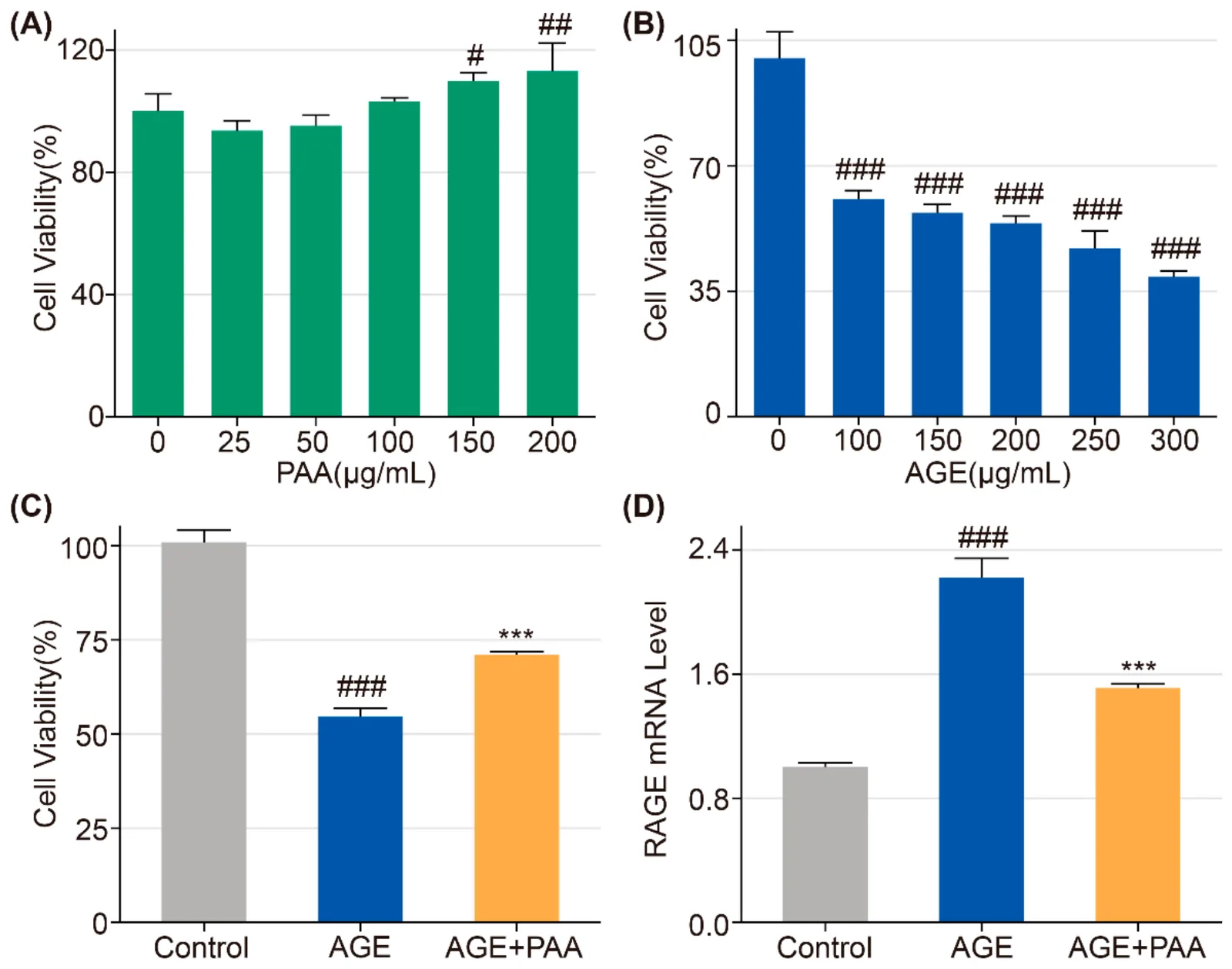
Effects of poricoic acid A (PAA) on cell viability and RAGE mRNA expression in AGE-induced MPC5 cells. (**A**) Cell viability of MPC5 cells at different concentrations of PAA. (**B**) Cell viability of MPC5 cells under varying AGE concentrations. (**C**) Effects of 100 μg/mL PAA on cell viability in 200 μg/mL AGE-induced MPC5 cells. (**D**) Levels of RAGE mRNA expression in MPC5 cells, AGE-induced MPC5 cells, and AGE-induced MPC5 cells treated with PAA. Data are presented as mean ± SEM from three independent experiments (*n* = 3). ^#^ *p* < 0.05, ^##^ *p* < 0.01, ^###^ *p* < 0.001 compared with the control MPC5 cells group; *** *p* < 0.001 compared with the AGE-induced MPC5 cells group.

To determine the optimal concentration of PAA, CCK-8 assays were performed. MPC5 cells were treated with PAA at concentrations of 0, 25, 50, 100, 150, and 200 μg/mL. As shown in Figure 5A, the cell viability values were (100.0 ± 1.7)%, (93.5 ± 1.5)%, (95.2 ± 1.4)%, (103.3 ± 1.3)%, (109.8 ± 1.2)%, and (113.1 ± 4.0)%, respectively. Notably, 100 μg/mL of PAA resulted in no clear cytotoxicity or proliferative effect on MPC5 cells (*p* = 0.875). Thus, this concentration was selected to further explore its pharmacological effects.

#### 3.6.2. Effect of PAA on AGE-Induced MPC5 Cells

The effect of AGE on MPC5 cell viability was assessed. When MPC5 cells were exposed to AGE at concentrations of 0, 100, 150, 200, 250, and 300 μg/mL, the cell viabilities were (100.0 ± 2.0)%, (60.7 ± 0.8)%, (57.0 ± 0.6)%, (53.9 ± 0.8)%, (46.9 ± 2.0)%, and (39.0 ± 0.7)%, respectively (Figure 5B). Of these concentrations, 200 μg/mL AGE induced a moderate cellular injury; therefore, this concentration was selected for subsequent experiments. As shown in Figure 5C, 100 μg/mL PAA significantly increased cell viability by (25.2 ± 0.3)% compared with that of the AGE-injured group (*p* < 0.0001).

#### 3.6.3. Effect of PAA on RAGE mRNA Expression in AGE-Stimulated MPC5 Cells

As shown in Figure 5D, *RAGE* mRNA was expressed at a low level in the MPC5 cells group, whereas its expression was significantly upregulated in AGE-induced MPC5 cells (*p* < 0.0001). Clearly, PAA treatment significantly decreased *RAGE* mRNA expression in AGE-stimulated MPC5 cells (*p* < 0.0001). These findings suggest that PAA inhibited AGE-induced upregulation of RAGE expression.

#### 3.6.4. Effect of PAA on the Downstream PI3K/AKT and NF-κB Pathways in the AGE/RAGE Axis

**Figure 6:**
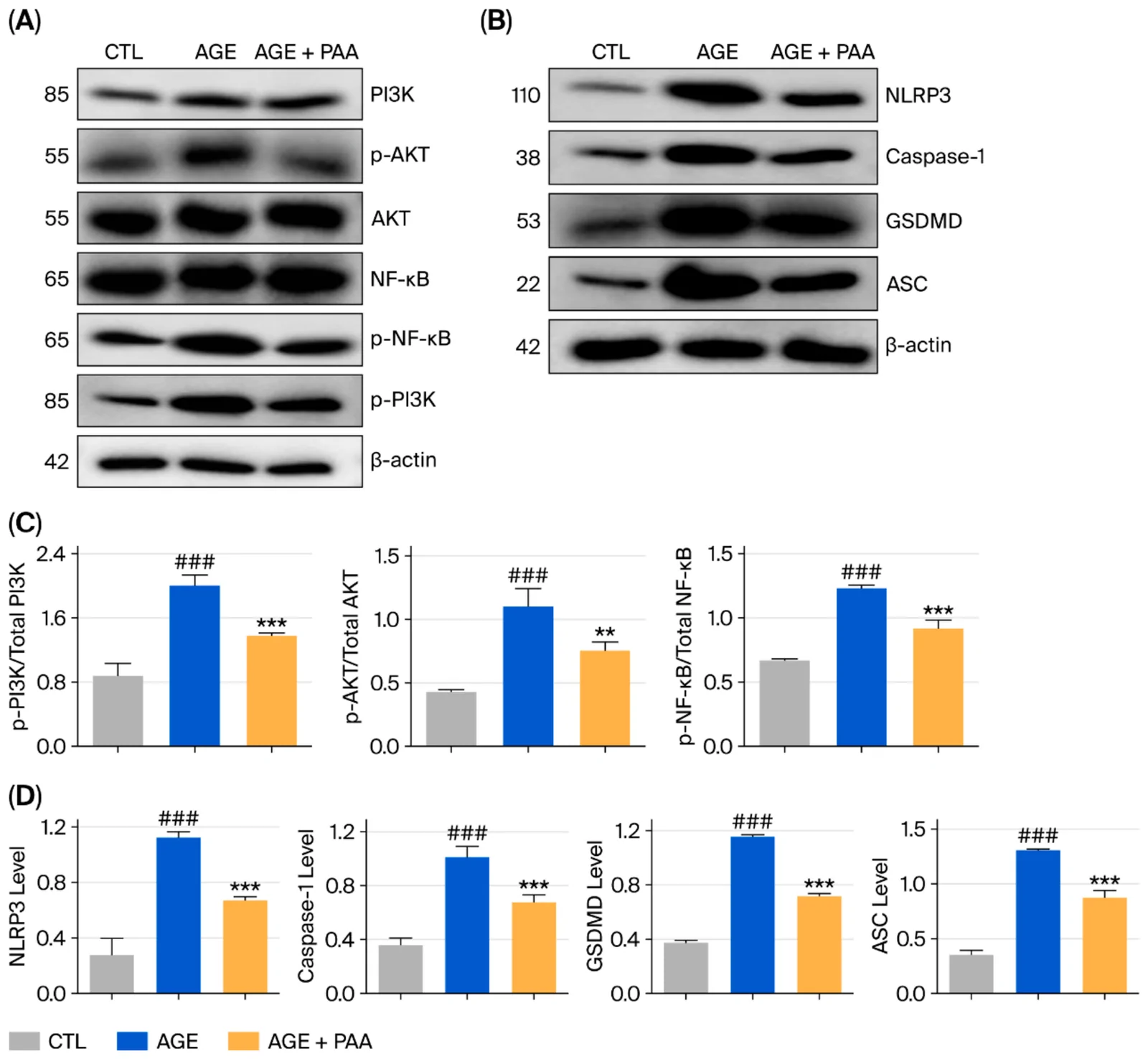
Effects of PAA on PI3K/AKT/NF-κB and NLRP3/caspase-1/GSDMD signaling pathways in AGE-induced MPC5 cells. (**A**) Representative blots showing total protein and phosphorylated protein levels of PI3K, AKT, and NF-κB in control MPC5 cells, AGE-induced MPC5 cells, and AGE-induced MPC5 cells treated with PAA. (**B**) Representative blots showing the protein levels of NLRP3, caspase-1, GSDMD, and ASC in the three groups. (**C**) The ratio of phosphoprotein to total protein (p-PI3K/PI3K, p-AKT/AKT, p-NF-κB/NF-κB) in the three groups. (**D**) Quantitative analysis of the protein levels of NLRP3, caspase-1, GSDMD, and ASC in the three groups. Data are presented as the mean ± SEM from three independent experiments (*n* = 3). ^###^ *p* < 0.001 compared with the MPC5 cells group; ** *p* < 0.01, *** *p* < 0.001 compared with the AGE-induced MPC5 cells group.

The effects of PAA on the expression of downstream signaling molecules in the AGE/RAGE pathway, specifically PI3K/AKT and NF-κB, were further investigated in vitro. Western blot results revealed that AGE stimulation significantly elevated the protein expression levels of p-PI3K, p-AKT, and p-NF-κB in MPC5 cells, with corresponding adjusted *p*-values provided in Supplementary Table S2, whereas these increases were reversed upon PAA treatment (Figure 6A,B).

#### 3.6.5. Effect of PAA on NLRP3 Inflammasome-Related Components

It is well-established that activation of the NF-κB signaling pathway could upregulate the transcription of inflammasome-related components. Accordingly, we further investigated the effect of PAA on protein expression of NLRP3 inflammasome-related components. As illustrated in Figure 6C,D, the protein expression levels of NLRP3, caspase-1, GSDMD, and ASC were markedly upregulated in AGE-induced MPC5 cells compared with the control group, with corresponding adjusted *p*-values provided in Supplementary Table S2. Importantly, PAA treatment exerted an inhibitory effect on the expression of these proteins. Collectively, these results suggest that PAA suppresses protein expression of NLRP3 inflammasome-related components, which may contribute to the alleviation of inflammation in DKD.

## 4. Discussion

DKD is a progressive chronic kidney disease primarily caused by hyperglycemia. Excessive accumulation of AGE and subsequent activation of its receptor RAGE act as critical mediators in driving the initiation and progression of DKD [24]. PC has exhibited promising efficacy for ameliorating DKD; nevertheless, its precise protective mechanisms remain incompletely elucidated. Therefore, the present study aimed to clarify the protective effects and underlying mechanisms of PC against DKD through integrating network pharmacology analysis combined with in vitro experimental validation using AGE-induced MPC5 cells.

The target of ingredients–network diagram demonstrated that 15 active compounds from PC interacted with 68 overlapping targets. For this set of 15 candidate compounds, it should be noted that they were screened by empirical OB ≥ 30% and DL ≥ 0.18 cut-offs from TCMSP. These thresholds are not customized for the present study and may exclude some potentially active ingredients. The moderate STRING threshold (0.4) applied in STRING-based PPI analysis may introduce potential false-positive interactions. Despite these methodological limitations, enrichment analysis as an exploratory tool was applied to generate testable hypotheses regarding the pharmacological mechanism of PC against DKD.

KEGG enrichment analysis revealed that the AGE-RAGE signaling pathway was the most significantly enriched pathway among the key targets, with the lowest *p*-values and a relatively high gene ratio. RAGE, as a pattern recognition receptor, can activate the innate immune system and promote the secretion of multiple inflammatory mediators [25]. Aberrant activation of the AGE-RAGE pathway contributes to DKD progression by triggering oxidative stress and inflammation, and targeting RAGE and its downstream signaling has emerged as a promising intervention strategy for DKD [26,27,28]. Li et al. found that Shenfu injection exerts an inhibitory effect on neuroinflammation in ischemic brain injury, primarily through suppression of the RAGE-PI3K-AKT signaling axis [29]. Additionally, excessive ROS generation induced by AGE-RAGE interaction triggers NF-κB-dependent inflammatory cascades and aggravates renal injury in DKD [30]. Consistent with the above findings, our results suggested that modulating the AGE-RAGE cascade may partly explain the reno-protective ability of the PC.

The network diagram of the “active ingredient–potential target–pivot pathway” revealed that triterpenoid components (including PAA, poricoic acid C, trametenolic acid, etc.) served as the primary active constituents. The core targets with high-degree values in the network were identified as *PIK3CA*, *AKT1*, *TNF*, *MAPK3*, and *MAPK8*. Notably, a previous network-pharmacology study of Qidan Dihuang Granule treating screened DKD showed that AGE-RAGE, PI3K-AKT, MAPK, TNF, EGFR, and STAT3 are key signaling nodes [31]. They exhibited considerable overlap with our top-five hub targets, supporting the reliability of our computational prediction. Among these, *PIK3CA* encodes the p110α catalytic subunit of the PI3K complex, and *AKT1* acts as the core downstream kinase of PI3K to regulate cellular proliferation and survival [32]. Beyond the PI3K/AKT cascade, TNF-mediated inflammation and ERK/JNK stress signaling, encoded by *MAPK3* and *MAPK8*, are well-known drivers of AGE-induced cell injury under diabetic conditions [33,34]. Pharmacological studies have demonstrated that natural bioactive compounds can alleviate DKD via modulating the PI3K/AKT cascade [35,36]. Consistent with these published findings, our predicted results suggested that the PI3K/AKT axis, together with inflammatory and stress-related signaling, is involved in the reno-protective effects mediated by PC against DKD.

However, network-pharmacology results may carry inherent database bias. More importantly, target screening based on network centrality tends to prioritize universal hub genes rather than strictly DKD-specific therapeutic targets. Nevertheless, these pharmacological findings still lay a foundation for further exploring the protective effect of PC against AGE-induced podocyte injury, a key pathological event in DKD. Molecular docking results further indicate that these bioactive ingredients, such as PAA, poricoic acid C, trametenolic acid, dehydroeburicoic acid, and cerevisterol, may have a favorable binding propensity toward the above core targets.

Given that PAA is one of the main active components of PC, and the AGE-RAGE signaling acts as an upstream trigger amplifying inflammatory cascades in DKD, we further experimentally evaluated key signaling nodes including *RAGE* mRNA, the PI3K/AKT pathway, NF-κB, and the NLRP3 inflammasome in AGE-induced MPC5 cells. This selection of signaling nodes is supported by the existing literature. First, PAA has exhibited favorable reno-protective bioactivities in previous studies [37,38]. Second, targeting the AGE/RAGE signaling pathway has been shown to ameliorate podocyte apoptosis in DKD [39]. Additionally, relevant studies have shown that geniposide can directly block AGE/RAGE signal transduction by inhibiting RAGE activation, thereby mitigating inflammatory responses [7]. Together, these findings provided a sound basis for us to explore the AGE-RAGE pathway, especially the modulatory effect of PAA on this signaling axis under DKD-mimicking conditions.

The cellular experimental results indicate that PAA exerts a protective effect against AGE-induced injury in MPC5 cells. Specifically, PAA suppressed the overexpression of *RAGE* mRNA, suggesting that PAA may modulate the AGE-RAGE signaling pathway. Notably, RAGE alterations at the mRNA level may not fully represent its protein abundance, and RAGE protein abundance remains to be measured in subsequent investigations. PAA also downregulated phosphorylation levels within the PI3K/AKT/NF-κB signaling cascade. Meanwhile, PAA suppressed protein expression of NLRP3 inflammasome-related components, accompanied by reduced levels of downstream pyroptosis-associated proteins. Only the total protein abundance of NLRP3 inflammasome-related molecules was determined, whereas their activated cleaved products were not examined. Additional functional verification is needed to confirm NLRP3 inflammasome activation. Taken together, these results suggest that PAA, as a major active constituent of PC, may confer the reno-protective effects of renal podocytes by regulating the AGE-RAGE axis and its downstream PI3K/AKT/NF-κB and NLRP3 signaling networks. However, the number of biological replicates was limited, and the assumptions for parametric ANOVA could not be fully verified. Furthermore, all pharmacological assessments of PAA in the present study were performed at the 200 μg/mL AGE-induced injury. Without examination of the reproducibility of PAA-mediated protection under less severe cellular injury induced by lower AGE concentrations, it remains difficult to distinguish protection against a DKD-relevant cellular phenotype from protection against severe nonspecific cytotoxicity. This limitation implies that a series of lower AGE-concentration experiments should be carried out in follow-up studies. In addition, only PAA was subjected to experimental validation in this study. Other triterpenoid constituents in PC have also been reported to possess biological activities [40]. Some of these components might not be among the nine candidate active compounds identified by network pharmacology analysis. Accordingly, other bioactive triterpenoid and highly ranked compounds await further experimental testing. Despite these limitations, from a therapeutic perspective, targeting this AGE-RAGE-NF-κB-NLRP3 signaling axis could represent a promising strategy direction for DKD-associated podocyte injury.

Although these findings are promising, several limitations of the present study should be acknowledged. First, all mechanistic findings in this study were obtained from an in vitro MPC5 cell model without cross-model validation of alternative podocyte lines and in vivo animal validation. Additionally, the NLRP3-related analysis was restricted to the detection of total proteins instead of its activated cleaved products. Furthermore, conclusive causal evidence supporting the AGE-RAGE axis remains incomplete because RAGE genetic manipulation assays (knockdown, overexpression, and functional rescue experiments) were not performed. These constitute inherent drawbacks of the present investigation. Second, the in vivo metabolic fate of PAA has not yet been fully elucidated. LC-MS-based metabolomics analysis would be helpful to clarify its metabolic pathways, intermediate metabolites, and bioavailability. Third, it remains to be experimentally validated whether the other PC-derived candidates screened by network pharmacology can also exert biological activities. Fourth, even if such protective activities exist, the therapeutic potency and precise signaling mechanisms remain poorly understood.

## 5. Conclusions

This study partially revealed the molecular mechanisms underlying the therapeutic effects of PC against DKD by integrating network pharmacology, molecular docking, and AGE-induced MPC5 cell experiments. Hub targets including *PIK3CA*, *AKT1*, *TNF*, *MAPK3,* and *MAPK8* were identified, and the AGE-RAGE signaling pathway was predicted to be the core regulatory pathway. In AGE-induced MPC5 cells, PAA decreased *RAGE* mRNA levels and suppressed downstream molecules (PI3K/AKT and NF-κB) within the AGE-RAGE signaling cascade. Additionally, PAA inhibited protein expression of NLRP3 inflammasome-related components. These results offer experimental evidence at the cellular level for further mechanistic exploration into PC against AGE-induced podocyte injury relevant to DKD.

## Figures and Tables

**Table 1:** Core nodes within the interaction network, degree centrality (DC), betweenness centrality (BC), and closeness centrality (CC) based on the PC-ingredient–target–pathway network.

Node Name	Degree Centrality	Betweenness Centrality	Closeness Centrality
PIK3CA	19	0.0529	0.4892
AKT1	19	0.0470	0.4823
TNF	19	0.0933	0.5037
MAPK3	18	0.0561	0.4823
MAPK8	17	0.0601	0.4690
MAPK14	16	0.0300	0.4564
PTPN1	15	0.0419	0.4823
ESR1	14	0.0432	0.4564
AR	14	0.0331	0.4755
PPARA	12	0.0180	0.4172
NR3C1	12	0.0241	0.4626
PTPN11	11	0.0247	0.4387
CYP19A1	11	0.0221	0.4503
HMGCR	11	0.0231	0.4564
PPARG	11	0.0135	0.4024
GSK3B	10	0.0770	0.4224
FL1 ((2R)-2-[(3S,5R,10S,13R,14R,16R,17R)-3,16-dihydroxy-4,4,10,13,14-pentamethyl-2,3,5,6,12,15,16,17-octahydro-1H-cyclopenta[a]phenanthren-17-yl]-6-methylhept-5-enoic acid)	17	0.0613	0.4722
FL4 (Cerevisterol)	16	0.1221	0.4928
FL13 (Poricoic acid C)	14	0.0279	0.4416
FL2 (Trametenolic acid)	13	0.0277	0.4533
FL9 (3beta-Hydroxy-24-methylene-8-lanostene-21-oic acid)	13	0.0277	0.4533
FL10 (Pachymic acid)	13	0.0888	0.4533
FL11 (Poricoic acid A)	13	0.0361	0.4533
FL15 (Dehydroeburicoic acid)	13	0.0200	0.4304
FL8 ((2R)-2-[(5R,10S,13R,14R,16R,17R)-16-hydroxy-3-keto-4,4,10,13,14-pentamethyl-1,2,5,6,12,15,16,17-octahydrocyclopenta[a]phenanthren-17-yl]-5-isopropyl-hex-5-enoic acid)	10	0.0134	0.4096
Proteoglycans in cancer	13	0.0368	0.4474
AGE-RAGE signaling pathway in diabetic complications	12	0.0317	0.4416
EGFR tyrosine kinase inhibitor resistance	11	0.0285	0.4096
Endocrine resistance	10	0.0171	0.4198
TNF signaling pathway	10	0.0236	0.4250
C-type lectin receptor signaling pathway	9	0.0129	0.4198
Insulin resistance	9	0.0249	0.4474
Osteoclast differentiation	9	0.0178	0.4250

**Table 2:** Binding energy values of molecular docking interactions between the effective ingredients (FL1, FL2, FL4, FL8, FL9, FL10, FL11, FL13, and FL15) and target proteins (PIK3CA, AKT1, TNF, MAPK3, and MAPK8).

Molecule Name	Target				
	PIK3CA (6GVF)	AKT1 (4EKL)	TNF (1A8M)	MAPK3 (6GES)	MAPK8 (3PZE)
FL1((2R)-2-[(3S,5R,10S,13R,14R,16R,17R)-3,16-dihydroxy-4,4,10,13,14-pentamethyl-2,3,5,6,12,15,16,17-octahydro-1H-cyclopenta[a]phenanthren-17-yl]-6-methylhept-5-enoic acid)	−6.3	−7.5	−4.1	−5.8	−5.7
FL2 (Trametenolic acid)	−5.9	−7.5	−5.1	—	−6.3
FL4 (Cerevisterol)	−4.8	−7.6	−3.8	−2.2	−8.0
FL8 ((2R)-2-[(5R,10S,13R,14R,16R,17R)-16-hydroxy-3-keto-4,4,10,13,14-pentamethyl-1,2,5,6,12,15,16,17-octahydrocyclopenta[a]phenanthren-17-yl]-5-isopropyl-hex-5-enoic acid)	−6.1	−8.3	2.2	2.2	−7.6
FL9 (3beta-Hydroxy-24-methylene-8-lanostene-21-oic acid)	−6.2	−7.6	2.6	—	−7.2
FL10 (Pachymic acid)	−6.8	−8.7	—	—	−6.9
FL11 (Poricoic acid A)	−7.5	−8.9	2.2	—	−4.1
FL13 (Poricoic acid C)	−7.0	−9.0	2.4	—	−3.8
FL15 (Dehydroeburicoic acid)	−6.7	−8.2	−3.4	−8.0	−7.8

## Data Availability

The public datasets used in this study were obtained from TCMSP, PubChem, GeneCards, DisGeNET, SwissTargetPrediction, STRING, and the Protein Data Bank (see Section 2.2, Section 2.3, and Section 2.6). The experimental data generated from this work can be made available by the authors upon reasonable request.

## Supplementary Materials

The following supporting information can be downloaded at: https://www.mdpi.com/article/10.3390/nu18203432/s1, Table S1: Full information of 15 candidate compounds, including FL label, Mol ID, molecule name, PubChem CIDs, and other identifiers; Table S2: Tukey-adjusted *p*-values for all pairwise group comparisons after one-way ANOVA for Figure 5 and Figure 6; Table S3: Summary of compound targets, DKD targets and overlapping targets.
